# Inhibitors of HIV-1 and Cathepsin L Proteases Identified from the Insect Gall of *Hypericum kouytchense*

**DOI:** 10.3390/ph15121499

**Published:** 2022-11-30

**Authors:** Bo Wen Pan, Jun Wei Xiao, Su Mei Li, Xin Yang, Xia Zhou, Qing Wen Sun, Mei Chen, Shou Xia Xie, Meena Kishore Sakharkar, Jian Yang, Ying Zhou, Ying Wei

**Affiliations:** 1 College of Pharmacy, Guizhou University of Traditional Chinese Medicine, Guiyang 550025, China; 2 Department of Pharmacology, Shenzhen People’s Hospital (The Second Clinical Medical College, Jinan University, The First Affiliated Hospital, Southern University of Science and Technology), Shenzhen 518020, China; 3 College of Pharmacy and Nutrition, University of Saskatchewan, Saskatoon, SK S7N 5E5, Canada

**Keywords:** *Hypericum kouytchense*, active ingredient, UPLC–HRMS, HIV-1 protease, cathepsin L protease, molecular docking

## Abstract

*Hypericum kouytchense* Lévl is a semi-evergreen plant of the Hypericaceae family. Its roots and seeds have been used in a number of traditional remedies for antipyretic, detoxification, anti-inflammatory, antimicrobial and antiviral functions. However, to date, no bioactivity compounds have been characterized from the insect gall of *H. kouytchens*. In this study, we evaluated the antiviral activities of different extracts from the insect gall of *H. kouytchen* against cathepsin L, HIV-1 and renin proteases and identified the active ingredients using UPLC–HRMS. Four different polar extracts (HW, H30, H60 and H85) of the *H. kouytchense* insect gall exhibited antiviral activities with IC_50_ values of 10.0, 4.0, 3.2 and 17.0 µg/mL against HIV-1 protease; 210.0, 34.0, 24.0 and 30.0 µg/mL against cathepsin L protease; and 180.0, 65.0, 44.0 and 39.0 µg/mL against human renin, respectively. Ten compounds were identified and quantified in the *H. kouytchense* insect gall extracts. Epicatechin, eriodictyol and naringenin chalcone were major ingredients in the extracts with contents ranging from 3.9 to 479.2 µg/mg. For HIV-1 protease, seven compounds showed more than 65% inhibition at a concentration of 1000.0 µg/mL, especially for hypericin and naringenin chalcone with IC_50_ values of 1.8 and 33.0 µg/mL, respectively. However, only hypericin was active against cathepsin L protease with an IC_50_ value of 17100.0 µg/mL, and its contents were from 0.99 to 11.65 µg/mg. Furthermore, we attempted to pinpoint the interactions between the active compounds and the proteases using molecular docking analysis. Our current results imply that the extracts and active ingredients could be further formulated and/or developed for potential prevention and treatment of HIV or SARS-CoV-2 infections.

## 1. Introduction

Viral infection is when a virus invades the body and replicates inside host cells, then produces toxins and causes disease [1]. Antiviral drugs target different stages of viral invasion, including recognition, fusion, entry and genome proliferation [2,3,4]. Cathepsin L protease (Cat L PR) plays an important role in the viral entry of SARS-CoV-2 by activating the spike protein in endosome or lysosome, and has been regarded as a key antiviral drug design target for SARS-CoV-2.

It has been shown that SARS-CoV-2 replication resembles HIV assembly and similar functional proteins are employed [5,6]. Thus, HIV protease inhibitors could be effective against SARS-CoV-2. In the latest guidelines, lopinavir/ritonavir (LPV/r), a protease inhibitor approved for the treatment of HIV, SARS-CoV and MERS-CoV, is repurposed for the treatment of COVID-19 [7,8]. Furthermore, other HIV protease (HIV-PR) inhibitors, such as Nelfinavir, Atazanavir and Darunavir, exhibited potent inhibition of SARS-CoV-2 replication [3,4,9,10,11,12]. Although vaccination has been undertaken globally, the world is still facing a big challenge in reduced efficacy of the vaccines towards SARS-CoV-2 variant strains. Continued research and development of antiviral agents, for either prophylactic or treatment purposes, is critical to human health even when COVID-19 is transitioning from pandemic to endemic.

*Hypericum kouytchense* Lévl, “*Da Guo Lu Huang*” in Chinese, is an herbal plant used in traditional Chinese medicine. Its seeds and roots possess antipyretic, detoxification, anti-inflammatory, antimicrobial and antiviral functions and are suggested for the treatment of tumors, jaundice, dysentery, amenorrhea and irregular menstruation in the “List of Chinese Herbal Medicine in Guizhou”. Furthermore, water or ethanol extract of *H. kouytchense* is widely utilized by local people in preparing a decoction or medicinal bath for the treatment of hepatitis, sore throat, epithysitis and injuries [13]. Pilepić and Maleš analyzed the polyphenol content in 18 *Hypericum* species and found that *H. kouytchense* has the highest content of 16.88% [14]. Several lines of evidence showed that *Hypericum* species possess antiviral functions. The bioactive secondary metabolites in these species, such as xanthones, ketides and dibenzo-1,4-dioxane derivatives, exhibited antiviral activities against herpes simplex viruses [15]. Acylphloroglucinols from *H. sampsonii* showed potent inhibition against HIV with EC_50_ of 0.97~2.97 μM and selectivity index of 4.80~7.70 [16]. Biyouyanagin A, a prenylated acylphloroglucinol from *H. chinense*, selectively suppressed HIV replication in H9 lymphocytes with a therapeutic index (TI) value higher than 31.3 [17]. Naphthodianthrones, including hypericin and pseudohypericin, also possess antiviral activity against various enveloped viruses [18]. It is noteworthy that the antiviral activity of hypericin is enhanced upon exposure to light, and this activity employs multiple functional modes including inhibition of viral budding [18,19]. Since the insect gall of *H. kouytchense* exhibits a better profile for the anti-inflammatory, antiviral and anticancer functions than the seeds and roots, we undertook the current study to evaluate the inhibitory activities of four polar extracts and ten compounds from the insect gall of *H. kouytchense* Lévl. Seven compounds showed strong inhibitory activities against HIV-1 PR. However, only hypericin exhibited strong inhibition of Cat L PR. Hypericin and naringenin chalcone were identified to be the major protease inhibitors in the four extracts. Specifically, hypericin was present in high amounts and showed the most potent inhibitions against both proteases. To the best of our knowledge, this is the first study on the antiviral function of the *H. kouytchense* Lévl insect gall, and our results may help in developing novel dual protease inhibitors.

## 2. Results and Discussion

### 2.1. Inhibition of HIV-1 and Cat L PRs by H. kouytchense Insect Gall Extracts

The four extracts of the insect galls of *H. kouytchense* were evaluated for their inhibitory effects towards HIV-1 PR (aspartic protease) and Cat L PR (cysteine protease). As shown in Table 1, HW, H30, H60 and H85 were potent inhibitions of HIV-1 PR with respective IC_50_ values of 10.0, 4.0, 3.2 and 17.0 µg/mL. For Cat L PR, H30, H60 and H85 showed potent inhibition with IC_50_ values of 34.0, 24.0 and 30.0 µg/mL, respectively. However, HW only showed moderate inhibition of Cat L PR with an IC_50_ value of 210.0 µg/mL. The inhibitory effects of these extracts were much weaker than that of the positive control (cathepsin L inhibitor, IC_50_: 6.8 × 10^−4^ µg/mL). The most potent extract, H60, possessed less than 0.003% of the activity of the positive control. This implies that *H. kouytchense* insect gall extracts might be more selective towards inhibiting HIV-1 proteases over Cat L proteases. To evaluate the physiological toxicity of the extracts, we measured the inhibitory activities of these extracts towards another human aspartic protease, renin. H30, H60 and H85 exhibited potent inhibitions of renin with IC_50_ values of 65.0, 44.0 and 39.0 µg/mL, respectively. However, HW showed only moderate inhibition of renin with an IC_50_ value of 180.0 µg/mL. The inhibitory activities of these extracts were lower than that of the positive control (renin inhibitor, IC_50_: 0.9 µg/mL), with H85 having about 2.3% of the activity of the positive control. Thus, we may conclude that the inhibitory activities of *H. kouytchense* insect gall extracts for HIV-1 PR are better than those for Cat L PR, and the HW extract was low toxicity.

### 2.2. Characterization of Selective Compounds in the H. kouytchense Insect Gall Extracts by UPLC–MS and UV–Vis Methods

**Figure 1:**
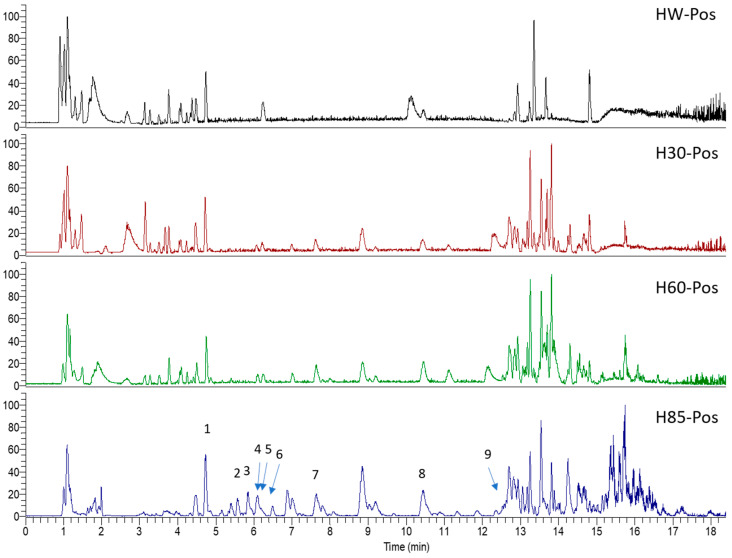
Ten selected active compounds in *H. kouytchense* insect gall extracts analyzed by UPLC–MS (positive mode).

**Figure 2:**
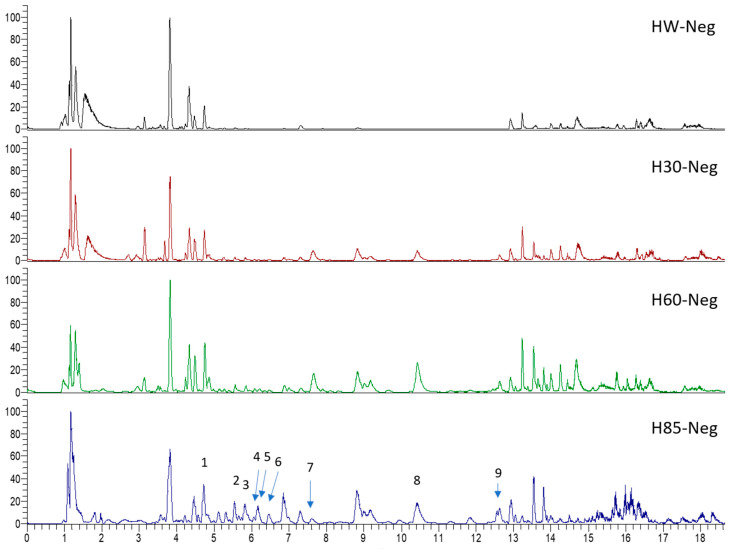
Ten selected active compounds in *H. kouytchense* insect gall extracts analyzed by UPLC–MS (negative mode).

*Hypericum* species contain a wide range of active ingredients, including prenylated acylphloroglucinols, meroterpenes, ketides, and dibenzo-1,4-dioxane derivatives [20]. Ten selected active compounds were profiled in the four extracts of *H. kouytchense* insect gall using UPLC–MS (Figure 1 and Figure 2 and Table 2). The 10 compounds were epicatechin, rutin, hyperoside, taxifolin-7-rhamnoside, quercetin-3-*O*-arabinose, quercitrin, eriodicytiol, quercetin, hypericin and naringenin chalcone. As shown in Table 3, epicatechin and naringenin chalcone were present in much higher quantities than the other compounds in the extracts. In addition, rutin, hyperoxide, taxifolin-7-rhamnoside, quercitrin, eriodicytiol and quercetin were present in concentrations higher than 5 μg/mg in H85, and quercetin, eriodicytiol and hypericin were present in concentrations higher than 9 μg/mg in H60.

### 2.3. Inhibition of HIV-1 and Cat L PRs by the Active Compounds from the H. kouytchense Insect Gall Extracts

To identify potential components responsible for the antiviral effects of *H. kouytchense* insect gall, we measured the inhibitory activities of the 10 active compounds against HIV-1 and Cat L PRs. As shown in Table 4 and Table 5, at a concentration of 1000.0 µg/mL, all compounds except rutin, quercetin and quercetin-3-arabinoside showed good inhibitions of HIV-1 PR, especially hypericin and naringenin chalcone, with IC_50_ values of 1.8 and 33.0 µg/mL, respectively. Towards Cat L PR, only hypericin, a dimeric anthraquinone, showed moderate inhibitory activity, with an IC_50_ value of 17,100.0 µg/mL. Thus, hypericin could be used as a lead compound in developing dual protease inhibitors with higher selectivity towards both HIV-1 and Cat L PRs.

### 2.4. Molecular Docking

**Figure 3:**
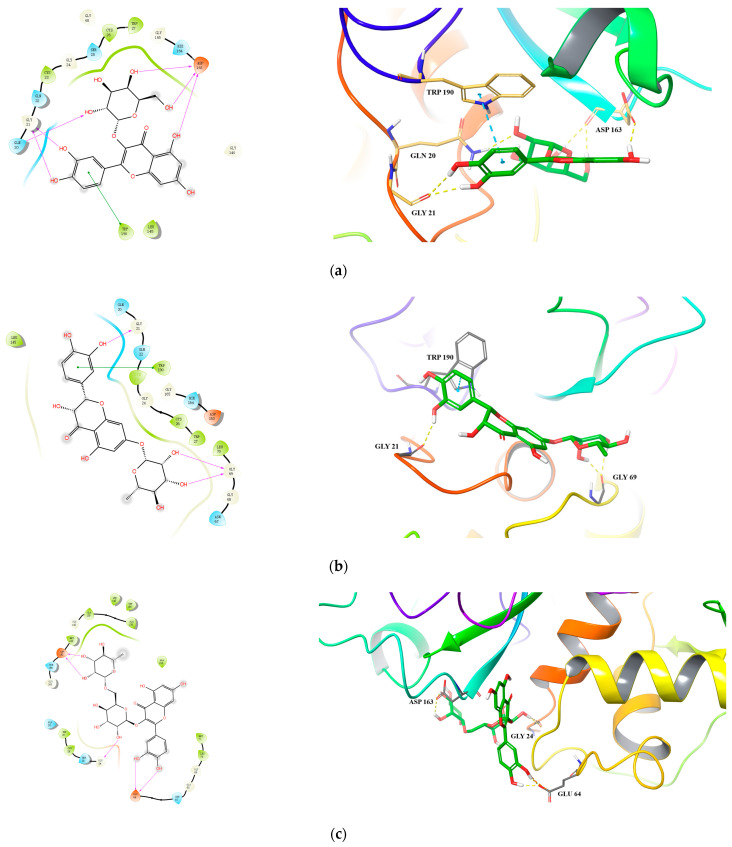

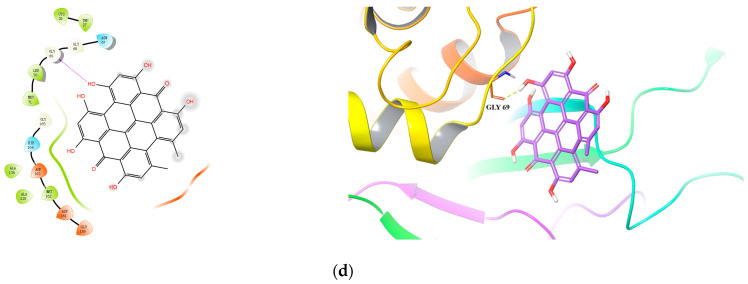
Predicted 2D and 3D binding models of cathepsin L protease with Hyperoside (**a**), Taxifolin-7-*O*-rhamnoside (**b**), Rutin (**c**) and Hypericin (**d**) by molecular docking.

For Cat L PR, we observed good binding affinity for six compounds with docking scores less than −6.0 and glide emodel values less than −50.0 kcal/mol, with hyperoside, taxifolin-7-*O*-rhamnoside and rutin being the top three (Table 6 and Figure 3). For hyperoside, six hydrogen bonds were formed with the active site residues Gly 21 and Asp 163. In addition, the benzene ring of hyperoside formed a π–π stacking with residue Typ 190 (Figure 3a). For taxifolin-7-*O*-rhamnoside, three hydrogen bonds were formed with residues Gly 69 and Gly 21 and a π–π stacking with residue Typ 190 (Figure 3b). For rutin, which had the lowest interaction energy of −79.958 kcal/mol, three hydrogen bonds were formed between the rutinoside group and residues Asp 163 and Gly 24 and two hydrogen bonds were formed between the 3’,4´-dihydroxyl of B ring and residue Glu 64 (Figure 3c).

**Figure 4:**
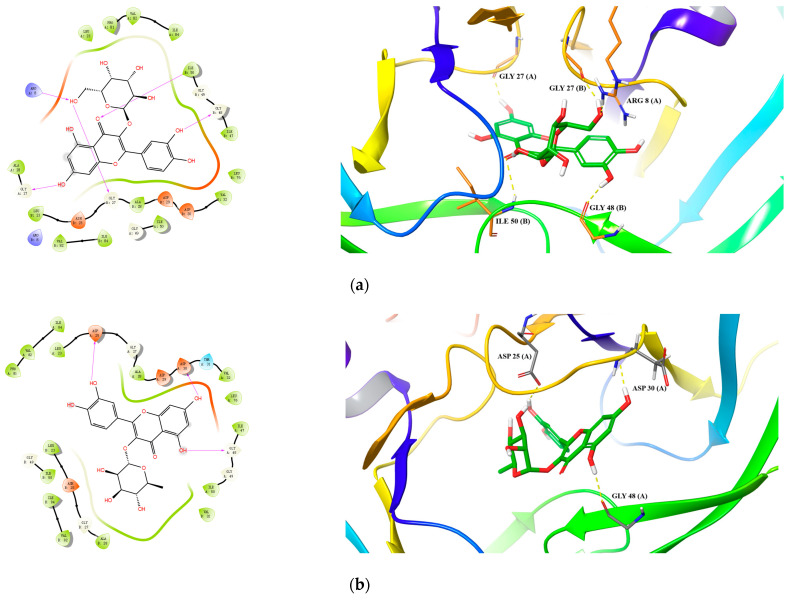

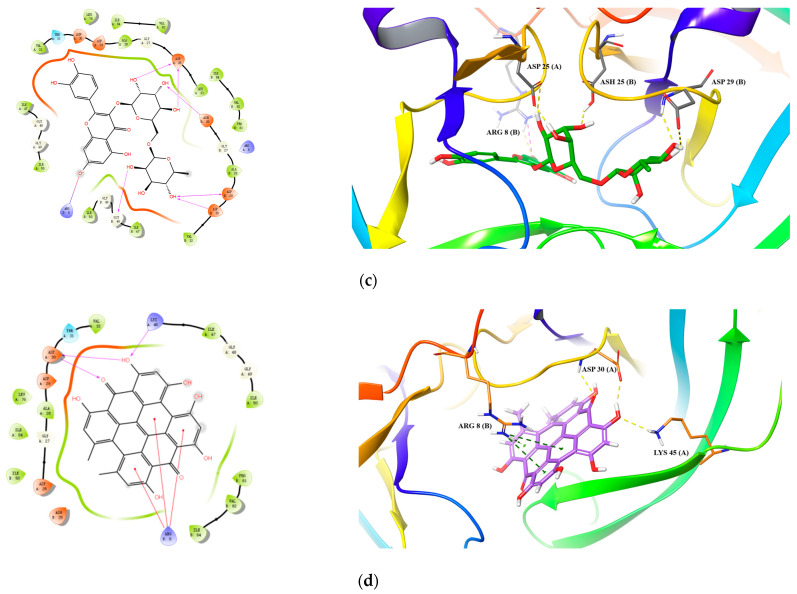
Predicted 2D and 3D binding models of HIV-1 Protease with Hyperoside (**a**), Quercitrin (**b**), Rutin (**c**) and Hypericin (**d**) through molecular docking.

For HIV-1 PR, all 10 active compounds provided reasonably good docking results (Table 7 and Figure 4). For hyperoside, its hydroxyls and carbonyls formed five hydrogen bonds with residues Arg 8(B), Gly 27(B), Gly 48(B) and Ile 50(B) (Figure 4a). For quercitrin, three hydrogen bonds were formed between its hydroxyl groups and residues Asp 25(A), Asp 30(A) and Gly 48(A) (Figure 4b). For rutin, we identified six hydrogen bonds between its hydroxyl groups and residues Asp 25(A), Ash 25(B), Gly 48(B), Asp 29(B) and Asp 30(B) and a salt bridge with Abg 8(B) (Figure 4c).

Specifically, for hypericin (the most potent compound against both HIV-1 and Cat L PRs), one hydrogen bond was formed between one of its hydroxyl groups and residue Gly 69 in Cat L PR (Figure 3d). Its hydroxyl and carbonyl groups formed four hydrogen bonds with residues Asp 30(A) and Lys 45(A) and a π–cation interaction between its benzene ring and residue Arg 8(B) in HIV-1 PR (Figure 4d). However, hypericin is sensitive to UV–Vis light and, thus, the docking results may not necessarily represent its real mechanism of action against HIV-1 and Cat L PRs. Further studies are warranted to identify the active form of hypericin and illustrate how it inhibits HIV-1 and Cat L PRs.

## 3. Materials and Methods

### 3.1. Plant Material

Insect gall of *H. kouytchense* was collected from Guiyang (Guizhou Province, China) in March 2020 and authenticated by Professor Qing Wen Sun, Guizhou University of Traditional Chinese Medicine (GUTCM). The voucher specimen (no. 202003/GLH) has been deposited at GUTCM.

### 3.2. Reagents and Instruments

Reference compounds (>98% purity), epicatechin, rutin, hyperoside, taxifolin-7-*O*-rhamnoside, quercetin-3-*O*-arabinose, quercitrin, eriodicytiol, quercetin, hypericin and naringenin chalcone, were purchased from Chengdu De Rui Ke Biological Technology Co., Ltd. (Chengdu, Sichuan Province, China). UPLC-QTOF-MS was performed on a Thermo Scientific UltiMate 3000 UHPLC system equipped with a Thermo Scientific Q Exactive Focus Orbitrap (Thermo Fisher Scientific, Bremen, Germany) with reagents CH_3_CN and HCOOH in LC–MS grade and propan-2-ol and CH_3_OH in HPLC grade. DMSO (>99.9% purity) for activity assay was purchased from Solarbio Co. Ltd. (Beijing, China). The fluorescence was detected using a BioTek Synergy II Microplate Reader (Biotec Co., Minneapolis, MN, USA).

### 3.3. Sample Preparation

Following our previously reported protocols [21], the air-dried insect gall of *H. kouytchense* Lévl was powdered and subsequently extracted three times with water or CH_3_OH–H_2_O (30:70, 60:40 and 85:15) for 1 h under reflux to obtain the HW, H30, H60 and H85 extracts, respectively. Concentration of the extract was 10 mg/mL for chemical profiling analysis and 100 mg/mL for quantitative data acquisition. All sample solutions were centrifuged at 14,000 rpm for 10 min before testing.

### 3.4. Fluorimetric HIV-1, Cat L and Renin PRs Inhibition Assay

The inhibitory activities against HIV-1, Cat L and Renin PRs were assayed by fluorimetric methods using our previously published protocols [21,22].

### 3.5. Characterization and Content Determination of the Main Active Components in the Extracts

Stock solutions (1.0 mg/mL) of reference compounds, epicatechin, rutin, hyperoside, taxifolin-7-rhamnoside, quercetin-3-*O*-arabinose, quercitrin, eriodicytiol, quercetin and naringenin chalcone, were prepared in methanol and stored at 4 °C. For each reference compound, an array of standard solutions was prepared from the stock by serial dilution with methanol with final concentrations of 100, 50, 25, 12.5, 6.25, 3.12, 1.56, 0.78 and 0.39 µg/mL. Stock solution of hypericin (10.0 mg/mL) was prepared in DMSO and stored at −80 °C. A series of solutions used for calibration were prepared in DMSO from the stock with respective final concentrations of 0.01, 0.02, 0.03, 0.05, 0.09, 0.1 and 0.2 mg/mL. Since hypericin is sensitive to light, both stock and calibration solutions were prepared without exposure to light. Chemical profiling of the four extracts (HW, H30, H60 and H85) was obtained with UPLC–Orbitrap–MS using our previously published protocols [21]. A UV–Vis–Microplate assay was applied for determination of hypericin by using a Synergy II microplate reader with detection wavelength of 588 nm. Sample solutions of HW, H30, H60 and H85 were prepared in water, 10% DMSO–water, 10% DMSO–water and 100% DMSO, respectively, with a final concentration of 10 mg/mL. The measurement was carried out in triplicate.

### 3.6. Docking Studies

The Schrödinger Suite 2021-1 and crystal structures of HIV-1 PR (PDB ID: 1QBS) and Cat L PR (PDB ID: 3OF9) were used in molecular docking analysis following our previously reported protocols [21,23].

## 4. Conclusions

SARS-CoV-2, the causative pathogen of COVID-19, is an enveloped, single-stranded RNA Betacoronavirus. As of 23 September 2022, it has infected more than 611 million people (confirmed cases) along with nearly 6.51 million deaths globally [24]. The current treatment options for COVID-19 mainly include antiviral drugs and immunotherapy. The genus of *Hypericum* has broad spectra of secondary metabolites with diverse biological activities. Previously, some bioactive compounds such as emodin, hypericin, hyperoside and/or isoquercitrin, chlorogenic acid, quercetin and quercitrin were observed in the leaves of *H. kouytchense* [25]. Sixteen chemical components were isolated from acrial parts of *H. kouytchense* Lévl, including xanthones, pentacyclic terpenoids, anthranone and phenolic acid [26]. However, there is no report on the chemical constituents and antivirus activity of *H. kouytchense* insect gall. In this study, we evaluated the inhibitory effects of four different extracts (HW, H30, H60, H85) of *H. kouytchense* insect gall against HIV-1 and Cat L PRs. H60 exhibited the most potent activity against both HIV-1 and Cat L PRs with IC_50_ values of 3.2 ± 2.97 µg/mL and 24.0 ± 1.44 µg/mL, respectively. Polarity of this fraction is likely to be similar to the most polar fraction of chloroform extracts of *H. perforatum*, in which active compounds have been identified to inhibit HIV infection [27]. Furthermore, we analyzed the contents of 10 active components in the extracts and measured their respective inhibitory activities against HIV-1 and Cat L PRs. Hypericin, which was highly present (0.1~1.17%) in the extracts, showed the most potent inhibitory activity against both proteases. The content is consistent with that recorded in U.S. Pharmacopeia (0.1~0.3%) or European Pharmacopiea (0.08%). Furthermore, hypericin has been suggested to treat SARS-CoV-2 as it could bind to four different SARS-CoV-2 target sites: spike protein (−9.7 kcal/mol), M^pro^ (−10.2 kcal/mol), PL^pro^ (−7.8 kcal/mol) and RdRp (−7.6 kcal/mol) [28]. The second most potent compound is naringenin chalcone, which showed a potent inhibition of HIV-1 PR (IC_50_ = 33.0 ± 4.59 µg/mL). Naringenin chalcone is also the most abundant component in the extracts. To the best of our knowledge, this is the first study showing that naringenin chalcone possesses antiviral function. For the development of more potent antivirus inhibitors from the insect gall of *H. kouytchense,* further in-depth research will be carried out in our lab.

## Figures and Tables

**Table 1:** The IC_50_ values of *H. kouytchense* insect gall extracts against HIV-1, Cat L and renin PRs (*n* = 3).

Name	HIV-1 PR (µg/mL)	Cat L PR (µg/mL)	Renin PR (µg/mL)
HW	10.0 ± 6.78	210.0 ± 4.05	180.0 ± 6.01
H30	4.0 ± 1.35	34.0 ± 1.64	65.0 ± 1.27
H60	3.2 ± 2.97	24.0 ± 1.44	44.0 ± 2.88
H85	17.0 ± 5.10	30.0 ± 4.22	39.0 ± 3.91
PC1	0.17	-	-
PC2	-	6.8 × 10^−4^	-
PC3	-	-	0.9

PC1: Pepstatin A, positive control for HIV-1 protease. PC2: Cathepsin L inhibitor, positive control for cathepsin L protease. PC3: Renin inhibitor, Ac–HPFV–(Sta)–LF–NH_2_ (AnaSpec Inc., Fremont, CA, USA), positive control for renin protease.

**Table 2:** Properties of the 10 selected active compounds in the *H. kouytchense* insect gall extracts.

No.	R_t_ [min]	Name	Structures	Formula
1	4.74	Epicatechin	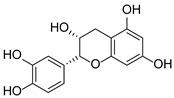	C_15_H_14_O_6_
2	5.47	Rutin	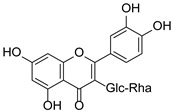	C_27_H_30_O_16_
3	5.73	Hyperoside	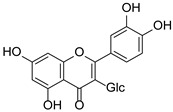	C_21_H_20_O_12_
4	6.05	Taxifolin-7-*O*-rhamnoside	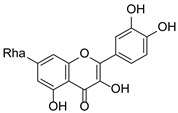	C_21_H_22_O_11_
5	6.37	Quercetin-3-*O*-arabinose	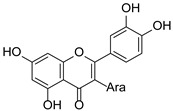	C_20_H_18_O_11_
6	6.73	Quercitrin	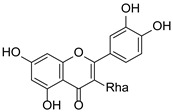	C_21_H_20_O_11_
7	7.52	Eriodicytiol	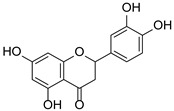	C_15_H_12_O_6_
8	10.45	Quercetin	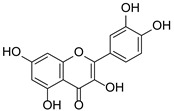	C_15_H_10_O_7_
9	12.57	Naringenin chalcone	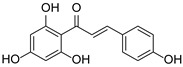	C_15_H_12_O_5_
10^※^		Hypericin	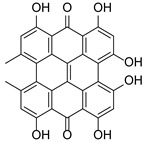	C_30_H_16_O_8_

^※^ Undetectable under current UPLC–MS condition.

**Table 3:** Content of the 10 active compounds in the extracts of *H. kouytchense* insect galls (µg/mg).

No.	Rt (min)	Name	HW	H30	H60	H85	Regression Equation
1	4.69	Epicatechin	82.19	124.26	114.07	89.45	Y = −1060.51 + 5929.96 × X R^2^ = 0.9995
2	5.47	Rutin	2.00	4.12	4.16	9.28	Y = 134859+341706 × X R^2^ = 0.9994
3	5.73	Hyperoside	1.01	3.08	3.61	10.77	Y = 195092+219026 × X R^2^ = 0.9992
4	6.05	Taxifolin-7-rhamnoside	1.30	1.96	1.93	5.12	Y = 216559+39301.7 × X R^2^ = 0.9983
5	6.37	Quercetin-3-*O*-arabinose	0.01	0.34	0.42	1.46	Y = 817687+540720 × X R^2^ = 0.9978
6	6.73	Quercitrin	0.82	3.16	3.61	14.05	Y = 52918.4+106963 × X R^2^ = 1.0000
7	7.52	Eriodicytiol	3.90	29.34	58.86	51.51	Y = 686143+95743.9 × X R^2^ = 0.9952
8	10.45	Quercetin	0.35	5.86	9.37	6.38	Y = −286872+268675 × X R^2^ = 0.9991
9	12.57	Naringenin chalcone	66.91	395.10	479.23	361.83	Y = 163887+175999 × X R^2^ = 0.9993
10^※^		Hypericin	1.49	4.03	11.65	0.99	Y = 4.5778 × X + 0.004 R^2^ = 1.000

^※^ Measured by microplate assay.

**Table 4:** Inhibition of Cat L PR by the 10 active compounds in *H. kouytchense* insect gall extracts (*n* = 3).

No.	Name	Cat L PR	
		Inhi% at 100.0 µg/mL	IC_50_ (µg/mL)
10	Hypericin	79.4 ± 2.02	17,100.0 ± 3.29
9	Naringenin chalcone	22.2 ± 6.77	>100,000.0
4	Taxifolin-7-*O*-Rhamnoside	18.1 ± 0.95	>100,000.0
6	Quercitrin	16.3 ± 3.24	>100,000.0
7	Eriodictyol	14.3 ± 2.32	>100,000.0
3	Hyperoside	11.8 ± 2.56	>100,000.0
5	Quercetin-3-*O*-Arabinoside	6.77 ± 0.46	>100,000.0
8	Quercetin	−3.67 ± 6.59	>100,000.0
2	Rutin	−4.81 ± 3.80	>100,000.0
1	Epicatechin	−17.7 ± 2.35	>100,000.0
PC	Cathepsin L inhibitor		6.8 × 10^−4^

**Table 5:** Inhibition of HIV-1 PR by the 10 active compounds in *H. kouytchense insect gall* extracts (*n* = 3).

No.	Name	HIV-1 PR	
		Inhi% at 1000.0 µg/mL	IC_50_ (µg/mL)
10	Hypericin	100.0 ± 7.87	1.8 ± 4.72
9	Naringenin chalcone	100.0 ± 1.96	33.0 ± 4.59
7	Eriodictyol	100.0 ± 4.98	190.0 ± 5.23
6	Quercitrin	94.4 ± 7.86	20.0 ± 5.04
3	Hyperoside	72.6 ± 1.40	39.0 ± 5.01
4	Taxifolin-7-*O*-rhamnoside	68.4 ± 5.45	550.0 ± 5.19
1	Epicatechin	66.0 ± 3.79	800.0 ± 2.72
8	Quercetin	50.3 ± 1.40	>100,000.0
5	Quercetin-3-*O*-arabinoside	32.6 ± 2.18	>100,000.0
2	Rutin	24.8 ± 3.85	>100,000.0
PC	Pepstatin A		0.17

**Table 6:** Docking of the 10 active compounds in the *H. kouytchense* insect gall extracts, along with the positive control cathepsin L inhibitor, against Cat L PR.

No.	Name	Docking Score	Glide Gscore	Glide Emodel (kcal/mol)
1	Hyperoside	−8.768	−8.796	−62.262
2	Taxifolin-7-*O*-rhamnoside	−8.572	−8.592	−60.924
3	Rutin	−8.558	−8.586	−79.958
4	Quercitrin	−6.920	−6.936	−53.793
5	Hypericin	−6.872	−6.932	−55.562
6	Quercetin-3-*O*-Arabinoside	−6.295	−6.324	−61.944
7	Eriodictyol	−5.737	−5.755	−44.473
8	Naringenin chalcone	−5.597	−6.337	−45.334
9	Quercetin	−5.528	−5.560	−41.583
10	Epicatechin	−5.403	−5.403	−41.822
PC	Cathepsin L inhibitor	−7.822	−7.823	−93.170

**Table 7:** Docking of the 10 active compounds in the *H. kouytchense* insect gall extracts, along with positive control pepstatin A, against HIV-1 PR.

No.	Name	Docking Score	Glide Gscore	Glide Emodel (kcal/mol)
1	Hyperoside	−11.349	−11.377	−73.883
2	Quercitrin	−10.691	−10.719	−87.889
3	Rutin	−10.578	−12.498	−94.728
4	Taxifolin-7-*O*-rhamnoside	−10.356	−10.376	−87.539
5.	Naringenin chalcone	−8.567	−8.823	−55.455
6	Quercetin-3-*O*-Arabinoside	−8.062	−9.982	−67.772
7	Quercetin	−7.782	−7.814	−60.119
8	Eriodictyol	−7.749	−7.768	−57.928
9	Epicatechin	−7.047	−7.047	−56.373
10	Hypericin	−6.222	−7.665	−54.973
PC	Pepstatin A	−10.940	−10.941	−131.591

## Data Availability

The data presented in this study are available in this article and on request from the author and corresponding author.

## Sample Availability

The samples of plant material, extraction and reference compounds of HK are available from the authors.

## Abbreviations

Cat L PR	cathepsin L protease
HIV PR	human immunodeficiency virus protease
HW	aqueous extract of *H. kouytchense* insect gall
H30	30% methanol extract of *H. kouytchense* insect gall
H60	60% methanol extract of *H. kouytchense* insect gall
H85	85% methanol extract of *H. kouytchense* insect gall
SARS-CoV-2	severe acute respiratory syndrome coronavirus 2
COVID-19	coronavirus disease 2019
HSV	herpes simplex virus
LC–MS	liquid chromatograph–mass spectrometer
DMSO	dimethyl sulfoxide
HPLC	high-performance liquid chromatography
UPLC–MS	ultra-performance liquid chromatography–mass spectrometry
GUTCM	Guizhou University of Traditional Chinese Medicine
